# Research on paclitaxel in chemotherapy-induced neuropathic pain: a bibliometric analysis integrating multiple databases

**DOI:** 10.3389/fpain.2026.1764444

**Published:** 2026-04-30

**Authors:** Rui Zeng, Min Xue, Mingxia Pan, Shuang Wang, Wenxi Xie, Weihong Ge, Han Xie

**Affiliations:** 1 Department of Pharmacy, Nanjing Drum Tower Hospital, School of Basic Medicine and Clinical Pharmacy, China Pharmaceutical University, Nanjing, Jiangsu, China; 2 School of Basic Medicine and Clinical Pharmacy, China Pharmaceutical University, Nanjing, Jiangsu, China; 3 Department of Pharmacy, Nanjing Drum Tower Hospital, Nanjing, Jiangsu, China; 4 School of Nursing, Fujian University of Traditional Chinese Medicine, Fuzhou, China

**Keywords:** bibliometric analysis, chemotherapy-induced peripheral neuropathy, CIPN, citespace, paclitaxel-induced neuropathic pain, research trends, VOSviewer

## Abstract

**Background:**

Paclitaxel-induced peripheral neuropathy (PIPN) significantly impairs the quality of life of cancer survivors. A systematic visualization of global research trends in this domain is currently lacking.

**Objective:**

This study aims to delineate the global research landscape, emerging hotspots, and evolutionary trends of PIPN research from **2000 to 2025**.

**Methods:**

Relevant literature was retrieved from Web of Science Core Collection and Scopus. Bibliometric analyses, including co-occurrence and thematic evolution, were performed using VOSviewer and CiteSpace.

**Results:**

A total of **1,795** eligible publications were analyzed, exhibiting an exponential growth trend (*R*^2^ = 0.9675*R*^2^ = 0.9675). The United States led with **718** publications and the highest citation impact. Supportive Care in Cancer was the most prolific journal. Research focus has shifted from initial toxicity descriptions to underlying mechanisms (e.g., oxidative stress) and quality of life. Cluster analysis identified emerging interests in “anxiety” and psychosocial comorbidities.

**Conclusion:**

PIPN research is rapidly expanding, moving from phenomenological observation to mechanistic elucidation and holistic management. Future directions include precision medicine and comprehensive biopsychosocial interventions.

## 1 Introduction

Advancements in oncological therapies have significantly prolonged cancer survival, yet treatment-related sequelae remain a critical challenge in survivorship care (1, 2). Among these, chemotherapy-induced peripheral neuropathy (CIPN) is a prevalent and dose-limiting adverse effect. Paclitaxel, a microtubule-stabilizing taxane essential for treating breast, ovarian, and lung cancers, is notoriously linked to high rates of neurotoxicity (3–5). Paclitaxel-induced peripheral neuropathy (PIPN) affects up to 80% of patients and is characterized by symmetrical survivors' quality of life (6). Unlike oxaliplatin-induced acute cold dysesthesia, PIPN often persists for years, creating a “painful survivorship” for which no FDA-approved prophylactic agents currently exist (7–10).

Despite the exponential growth in research on PIPN mechanisms—ranging from mitochondrial dysfunction to neuroinflammation—the global academic landscape remains fragmented (11–13). Existing reviews are predominantly qualitative or focus broadly on general CIPN, failing to capture the specific intellectual structure, evolving hotspots, and collaborative networks unique to paclitaxel-induced neurotoxicity (14–18, 36, 37).

To address this gap, this study provides the first comprehensive bibliometric analysis specifically focused on paclitaxel-induced neuropathic pain over the past 25 years (2000–2025). By integrating data from Web of Science and Scopus and utilizing VOSviewer and CiteSpace, we aim to: (1) visualize the global distribution of research forces; (2) delineate the evolution of research themes from toxicity description to mechanistic exploration; and (3) identify emerging frontiers such as psychosocial management. This quantitative mapping offers a robust scientific foundation for guiding future translational research and clinical strategies.

The study protocol has been preregistered on the Open Science Framework, with the doi: https://doi.org/10.17605/0SF.I0/GQCFA.

## 2 Data and methods

### 2.1 Data sources and search strategy

This study selected the Web of Science Core Collection (WoSCC) and Scopus as core data repositories, given their well-recognized advantages and complementary coverage scope in bibliometric research. The Web of Science Core Collection indexes over 12,000 high-impact academic journals across multiple disciplinary fields, and it is universally acknowledged as one of the most credible tools for conducting rigorous bibliometric investigations (19). Scopus, on the other hand, is recognized as the largest abstract and citation database for peer-reviewed scholarly literature, covering an extensive range of disciplinary domains (20). By integrating these two complementary databases, this study achieves exhaustive retrieval of the growing global scholarly output focusing on paclitaxel-induced neuropathic pain, while simultaneously minimizing the risk of literature omission. Such synergy between the two platforms helps alleviate potential retrieval bias, thereby significantly enhancing the representativeness and methodological rigor of the analytical findings (21). The search period was defined from January 1, 2000, to November 30, 2025. To ensure a comprehensive and systematic literature retrieval, the search strategy was meticulously designed based on prior relevant studies and aligned with the Medical Subject Headings (MeSH) vocabulary (22). This approach allowed for the identification of synonymous terms, semantic variations, and relevant concepts within the biomedical knowledge domain.

The specific search string employed was as follows: TS = (paclitaxel OR Taxol OR “Bris Taxol” OR NSC125973 OR Paxene OR Anzatax OR Onxol OR Praxel) AND TS = (CIPN OR “Chemotherapy- Induced Peripheral Neuropathy” OR “Peripheral Nervous System Disorder” OR “Peripheral Neuropath*” OR “PNS Disease” OR “Peripheral Nerve Disease” OR “Peripheral Nerve” OR Endoneurium OR Epineurium OR “neuropathic pain” OR allodynia OR hyperalgesia OR dysesthesia) AND TS = (cancer OR oncolog* OR Neoplas* OR Tumor OR Malignanc*) AND PY = 2000–2025 AND DT = (Article OR Review). To ensure the inclusion of high-quality, peer-reviewed scientific evidence, the document types were strictly restricted to “Articles” and “Reviews” (23).

### 2.2 Inclusion and exclusion criteria

Inclusion criteria: This study included all studies that investigated PIPN or CIPN with a specific focus on paclitaxel. Publications were considered eligible regardless of their research design, methodology, or publication type, provided that their core subject matter addressed paclitaxel- associated neurotoxicity (24).

Exclusion criteria: Literature that did not meet the aforementioned core requirements was excluded based on the following criteria:

(1) Irrelevant Subjects: Studies that did not involve paclitaxel or failed to address CIPN and its associated symptoms;
(2) Unrelated Topics: Research that was unrelated to neuropathic pain, peripheral neuropathy, or the neurological adverse effects specifically attributable to paclitaxel;
(3) Insufficient Information: Publications presenting insufficient information, including those with unclear incomplete data presentation; research objectives, inadequate descriptions of methodologies, or
(4) Non-Peer-Reviewed Publications: Documents that were not subject to peer review, such as conference abstracts, editorials, letters to the editor, and case reports lacking substantial research content;
(5) Language Restrictions: Articles published in languages other than English were excluded.

### 2.3 Screening strategy

To minimize selection bias and ensure data integrity, the initial screening of all retrieved records was executed independently by two separate investigators (RZ and WX). This process adhered strictly to the PRISMA 2020 (Preferred Reporting Items for Systematic Reviews and Meta-Analyses) guidelines (25). Agreements between reviewers were substantial (Cohen's Kappa > 0.85), and any discrepancies were resolved through consultation with a senior investigator (HX) to reach consensus (26, 27, 39). Non-English publications were excluded to maintain data consistency and comparability, as English serves as the universal language of scientific communication in this domain. Both original articles and reviews were included in the final dataset and analyzed collectively to capture both primary empirical evidence and comprehensive expert syntheses.

### 2.4 Methodologies and data analysis

Bibliometric analyses and visualization were conducted using VOSviewer (version 1.6.20) and CiteSpace (version 6.2.R6). VOSviewer was primarily utilized to generate co-occurrence and co-authorship networks. Key metrics included Total Link Strength (TLS), defined as the aggregate focus of collaborative or co-occurrence interactions assigned to a specific item (e.g., author, country, or keyword), reflecting its centrality within the network (28). CiteSpace was employed to perform burst detection and keyword clustering analysis (using Log-Likelihood Ratio, LLR) to identify research hotspots and temporal trends (29, 30, 38).

### 2.5 Ethical considerations

his study exclusively utilized data retrieved from publicly accessible academic databases. Given the absence of direct involvement of human participants in the research design, ethical approval for primary data collection was not required, and the acquisition of informed consent from participants was deemed unnecessary.

## 3 Results

### 3.1 Analysis of publication outputs

**Figure 1:**
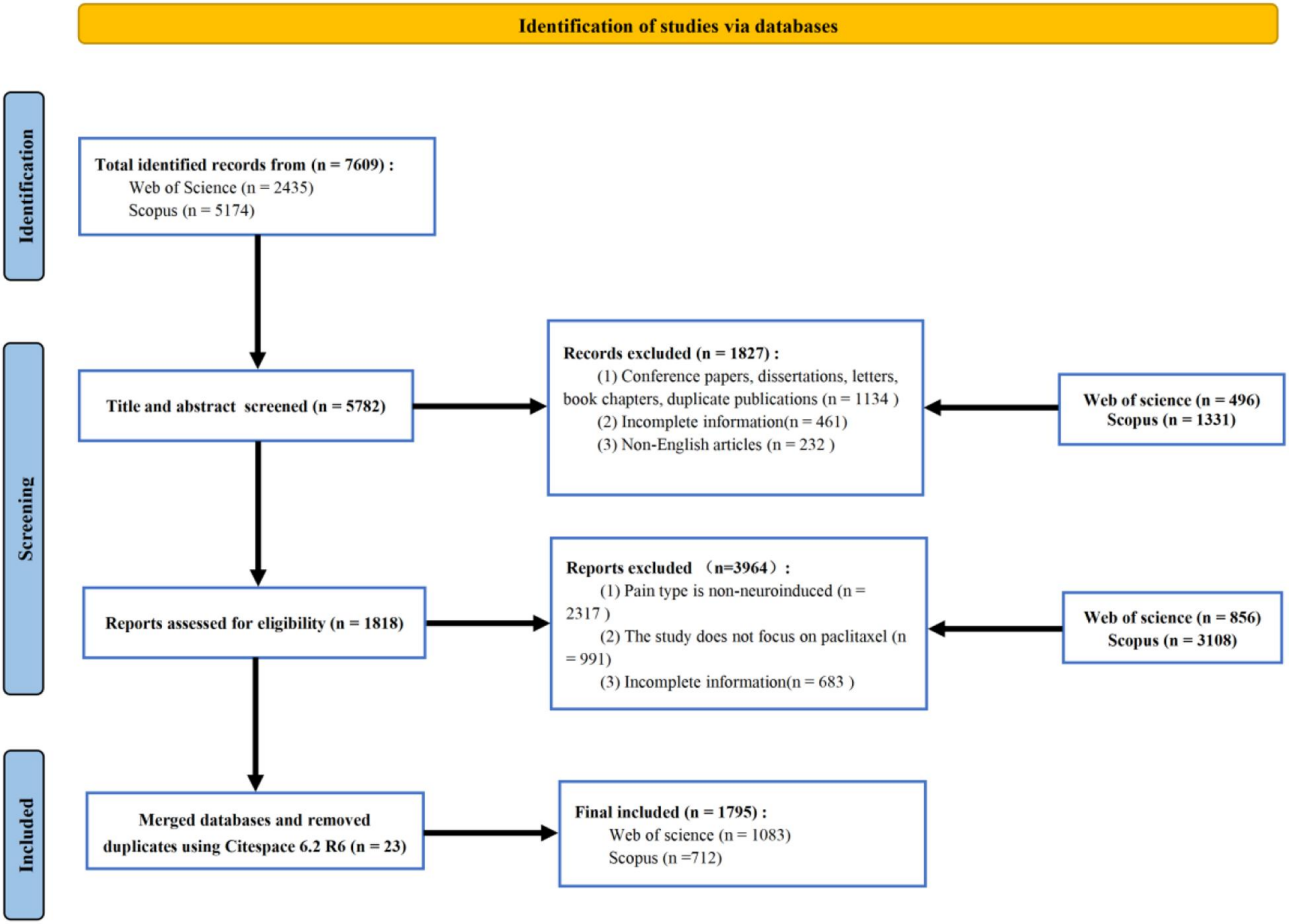
Flowchart of the literature-screening process.

**Figure 2:**
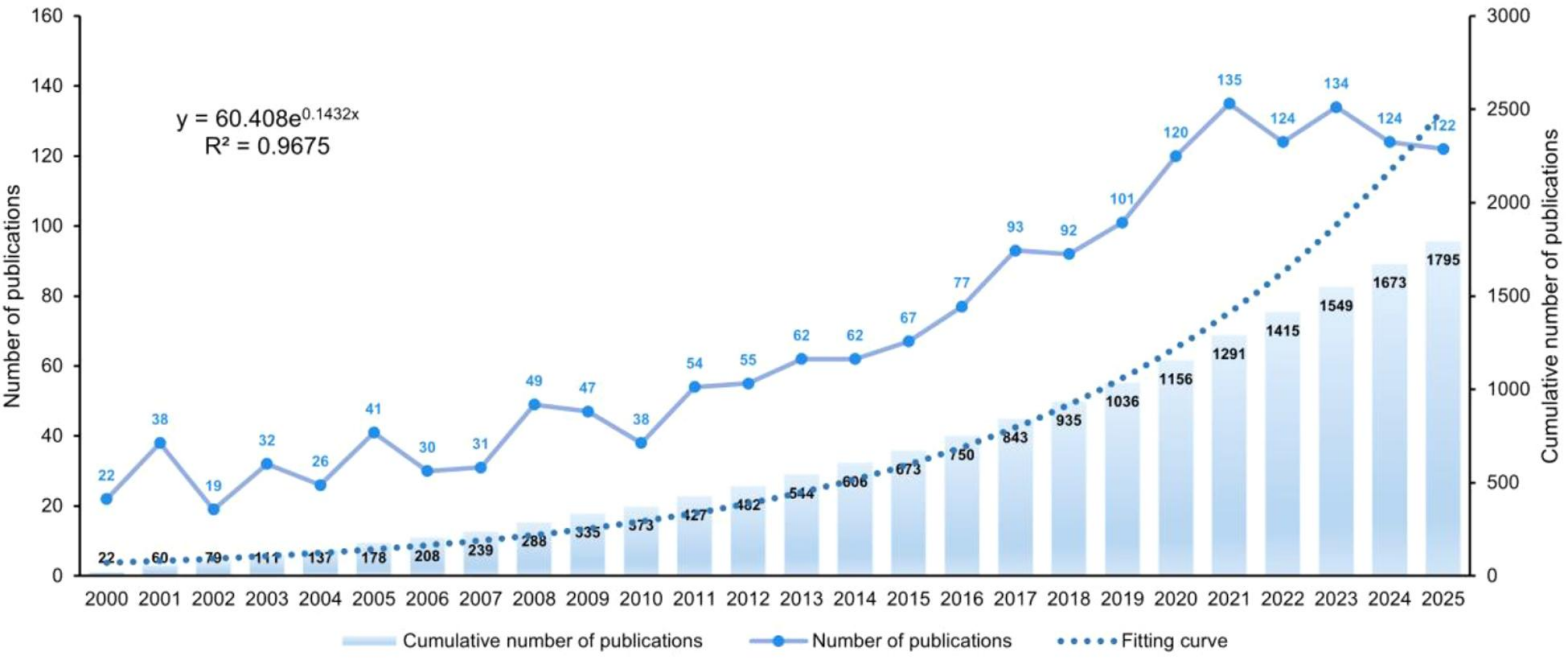
Number of publications by year (2000–2025).

Figure 1 presents the detailed workflow for literature selection in this bibliometric analysis. An initial comprehensive literature search was conducted in the Web of Science and Scopus databases, retrieving a total of 7,609 records, of which 2,435 were derived from Web of Science and 5, 174 from Scopus. After a preliminary evaluation, 1,827 records were excluded according to predefined criteria, including document type and language, resulting in 5,782 records eligible for title and abstract screening. During this screening stage, 3,964 articles were excluded, mainly due to the fact that the pain type examined was non-neuropathic or the studies did not specifically focus on paclitaxel. Subsequently, 1,818 full-text articles were evaluated for final eligibility. Following dataset merging and the removal of 23 duplicate records via CiteSpace software (31), a total of 1,795 unique publications were ultimately included in the analysis, with 1,083 from Web of Science and 712 from Scopus. Figure 2 illustrates the trends in annual and cumulative publication outputs in the field from 2000 to 2025. Overall, the field of paclitaxel-induced neuropathic pain has exhibited a steady and consistent upward trend in academic output. Annual publication output increased from a modest 22 articles in 2000 to a historic peak of 135 articles in 2021. Although slight fluctuations were noted between 2022 and 2024, with annual publication counts of 124, 134, and 124 respectively, the overall output has remained substantial. As of the data collection cutoff date (November 30, 2025), the publication count for 2025 has reached 122; however, this represents a partial year of data, and the final annual output is expected to be higher. Curve fitting analysis of the cumulative number of publications revealed a strong fit to an exponential growth model (y = 60.408e0.1432xy = 60.408e0.1432x), as evidenced by a high coefficient of determination (*R*^2^ = 0.9675*R*^2^ = 0.9675). This finding highlights the rapid expansion and active knowledge accumulation in this research field.

### 3.2 Analysis of countries/regions

**Figure 3:**
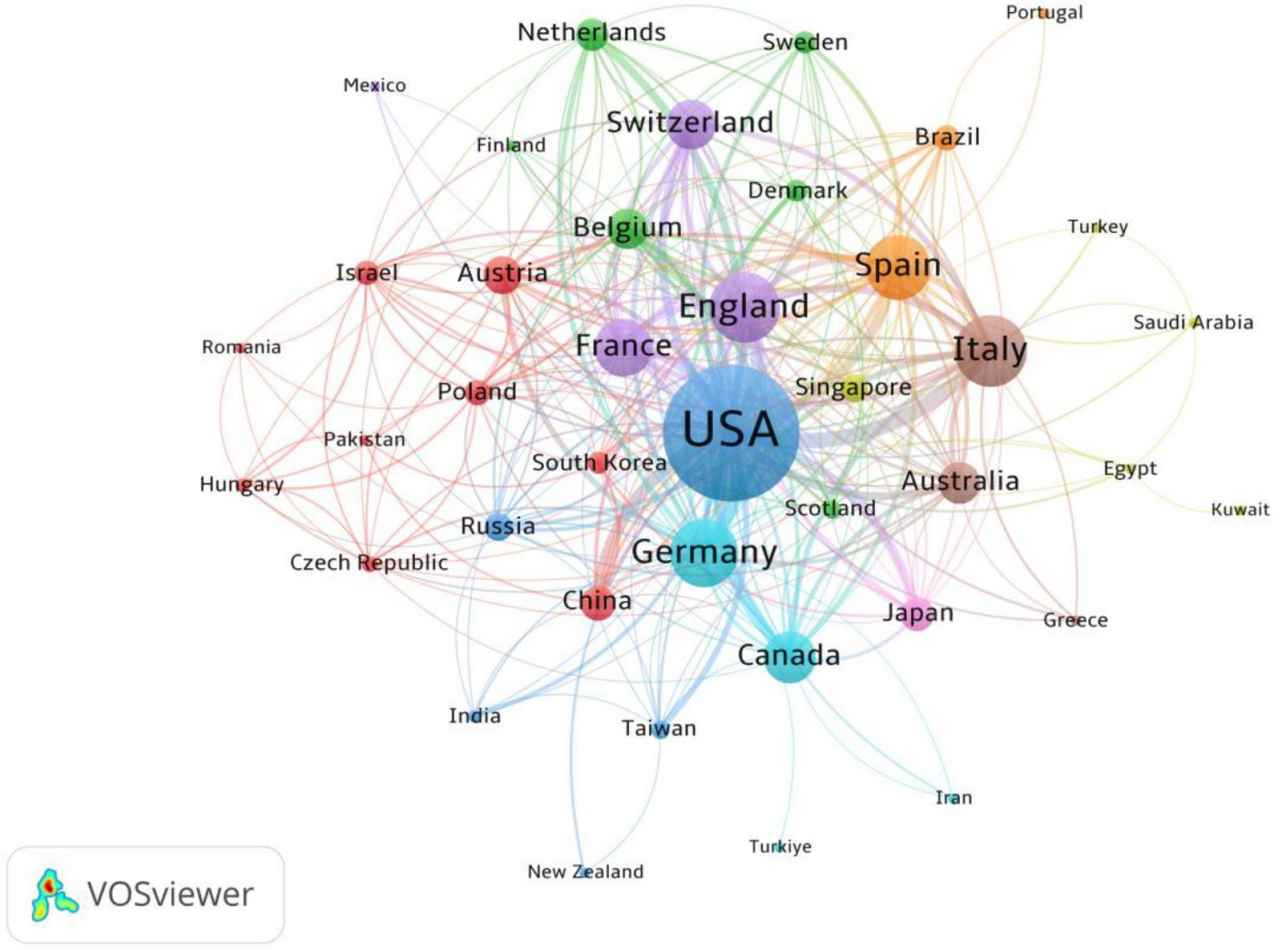
Visualization of research networks of countries/regions.

Research on paclitaxel-induced neuropathic pain has garnered contributions from a total of 80 countries and regions globally. Figure 3 illustrates the global geographical distribution of these scholarly outputs, which reveals a prominent concentration of research activity in North America, East Asia and Western Europe. Table 1 presents the top 10 countries ranked by publication output. The United States (USA) leads the field in terms of publication productivity, with 718 articles accounting for approximately 40% of all relevant publications worldwide. Japan and China rank second and third, respectively, with 226 and 207 publications. Notably, the USA excels not only in publication quantity but also in academic influence and collaborative density, boasting the highest Total Citations (TC) of 46,993 and a Total Link Strength (TLS) of 408. Although China and Japan are among the top three in terms of publication productivity, their Average Citations per Publication (Avg. Citations) are relatively modest, at 21.25 and 23.77, respectively. In contrast, Australia, despite ranking tenth in publication count with 57 articles, attains the highest average citation impact at 152.98. Similarly, Spain and Italy have also exhibited high research quality, with average citations of 113.61 and 83.87, respectivel.

**Table 1:** Top 10 countries ranked by number of publications.

Rank	Country/region	Articles (NP^a^)	Total citations (TC^b^)	TLS^c^	Avg. citations^d^
1	USA	718	46,993	408	65.45
2	Japan	226	5,372	56	23.77
3	China	207	4,398	59	21.25
4	Italy	138	11,574	162	83.87
5	South Korea	85	2,259	32	26.58
6	Germany	83	4,156	151	50.07
7	Spain	72	8,180	141	113.61
8	England	67	4,522	159	67.49
9	France	61	3,391	119	55.59
10	Australia	57	8,720	77	152.98

a NP, number of publications.

b TC, total citations.

c TLS, total link strength (from international co-authorship network).

d Avg. citations, average citations per publication.

### 3.3 Analysis of institutions

**Figure 4:**
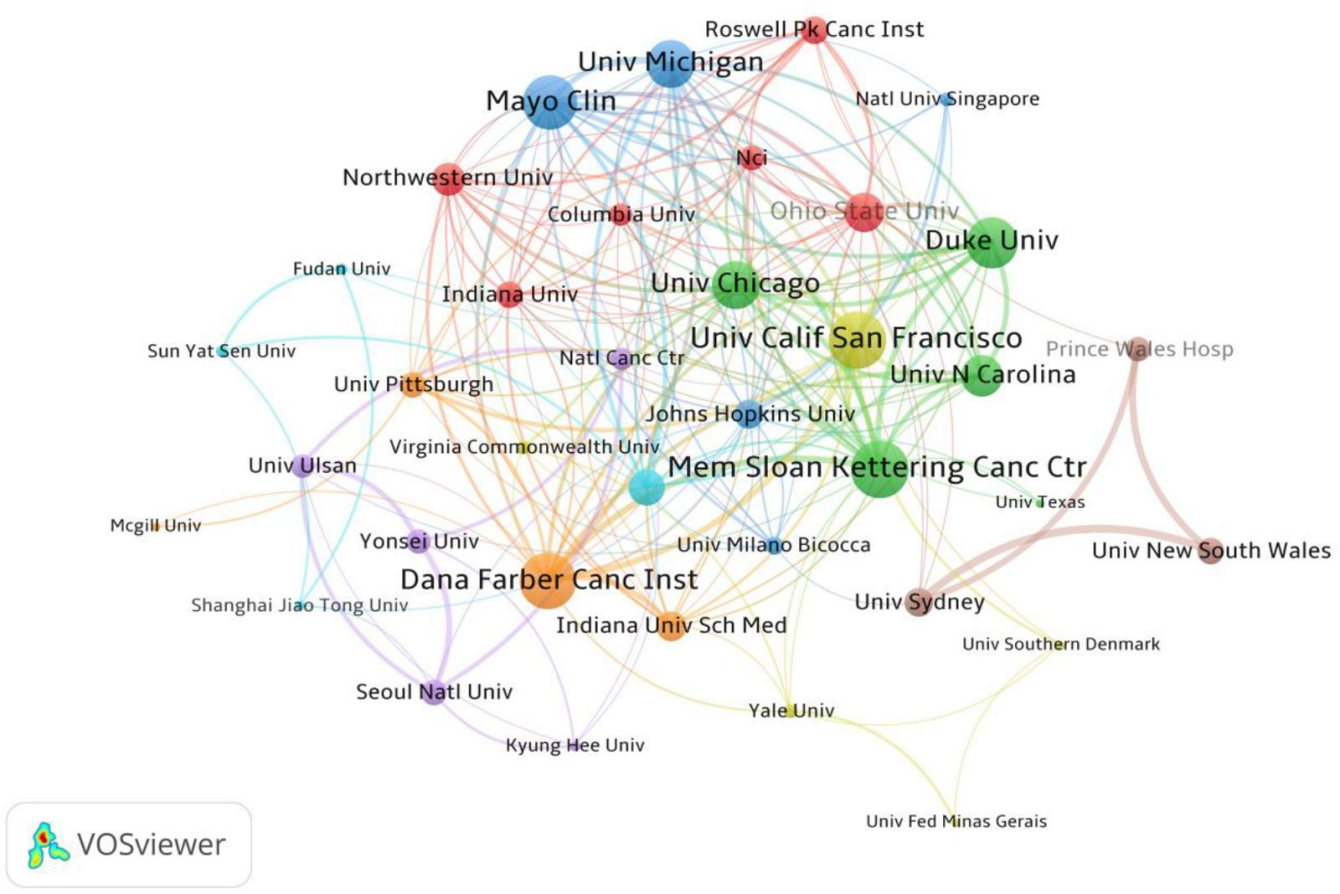
Visualization of research networks of institutions.

Globally, a total of 2,548 institutions have made substantial contributions to the field of paclitaxel- induced neuropathic pain. Figure 4 provides a visual representation of the collaborative network among key institutions with node size proportional to the number of publications and connecting lines indicating the strength of collaborative relationships. This visualization reveals several dense clusters of inter-institutional cooperation primarily led by top-tier cancer research centers and comprehensive universities in the United States. Table 2 delineates the top 10 institutions ranked by publication output. The University of Texas MD Anderson Cancer Center ranks first with 63 publications, solidifying its preeminent position in paclitaxel neurotoxicity research. Closely trailing are the Mayo Clinic and the University of Michigan with 54 and 45 publications respectively. Notably, nine out of the top 10 most prolific institutions are based in the United States. The University of Milano-Bicocca from Italy is the sole non-US representative, ranking sixth with 40 publications, which further confirms the dominant role of US institutions in this research area. Regarding citation impact, the National Cancer Institute (NCI) though ranking 8th in terms of publication output (33 articles) boasts the highest average citations per publication (90.85), a testament to the exceptional academic impact of its research. Similarly, the Dana-Farber Cancer Institute also exhibits significant academic impact with an average citation count of 82.84 per paper. In contrast, the National Cancer Center which ranks 9th in publication output exhibits a relatively lower citation impact with an average citations per publication of 25.43 despite its considerable publication volume.

**Table 2:** Top 10 institutions ranked by number of publications.

Rank	Country/region	Publications (NP^a^)	Total citations (TC^b^)	TLS^c^	Avg. citations^d^
1	Univ Texas Md Anderson Canc Ctr	63	4,478	42	71.08
2	Mayo Clin	54	3,222	74	59.67
3	Univ Michigan	45	2,405	63	53.44
4	Mem Sloan Kettering Canc Ctr	44	3,131	82	71.16
5	Univ Calif San Francisco	42	3,002	81	71.48
6	Univ Milano Bicocca	40	2,930	16	73.25
7	Dana Farber Canc Inst	38	3,148	78	82.84
8	Nci	33	2,998	25	90.85
9	Natl Canc Ctr	30	763	21	25.43
10	Duke Univ	28	2,219	69	79.25

a NP, number of publications.

b TC, total citations.

c TLS, total link strength (from institutional co-authorship network).

d Avg. citations, average citations per publication.

### 3.4 Analysis of journals

Table 3 enumerates the top 10 academic journals in the field of paclitaxel-induced neuropathic pain ranked by publication volume. These core journals span multiple disciplines, including oncology, pharmacology, pain management, and supportive care, which underscores the prominent multidisciplinary nature of this research topic. Supportive Care in Cancer ranked first as the most productive journal, with 60 relevant publications, reflecting the strong academic focus on the clinical management of chemotherapy-related side effects and the improvement of quality of life in cancer patients. Cancer Chemotherapy and Pharmacology took the second place with 44 publications, highlighting the crucial role of pharmacological research in this field. In terms of academic impact, the Journal of Clinical Oncology, although ranking third in publication volume with 40 articles, exhibited overwhelming dominance in citation metrics. It had a Total Citation (TC) count of 6,570 and an impressive Average Citations per Publication (Avg. citations) of 164.25, which significantly outperformed all other journals and solidified its status as the most authoritative source in this research area. Additionally, the journal Pain, ranking seventh in publication volume with 30 articles, had the highest Total Link Strength (TLS) of 683 and the second-highest average citation count (103.07). These findings highlight its pivotal role as a core hub for mechanistic and basic research in neuropathic pain. In contrast, Anticancer Research, which ranked eighth with 29 publications, exhibited relatively limited academic impact, with an average citation count of only 9.38 per article.

**Table 3:** Top 10 journals ranked by number of publications.

Rank	Journal	Articles (NP^a^)	Total citations (TC^b^)	TLS^c^	Avg. citations^d^
1	Supportive Care In Cancer	60	1,836	515	30.6
2	Cancer Chemotherapy And Pharmacology	44	1,035	211	23.5227
3	Journal Of Clinical Oncology	40	6,570	410	164.25
4	Clinical Cancer Research	40	2,530	440	63.25
5	Annals Of Oncology	38	2,056	173	54.1053
6	Breast Cancer Research And Treatment	37	1,783	315	48.1892
7	Pain	30	3,092	683	103.0667
8	Anticancer Research	29	272	83	9.3793
9	Cancer	28	1,733	240	61.8929
10	Gynecologic Oncology	28	1,013	94	36.1786

a NP, number of publications.

b TC, total citations.

c TLS, total link strength (from bibliographic coupling/cocitation network).

d Avg. citations, average citations per article.

### 3.5 Analysis of authors and coauthorship networks

**Figure 5:**
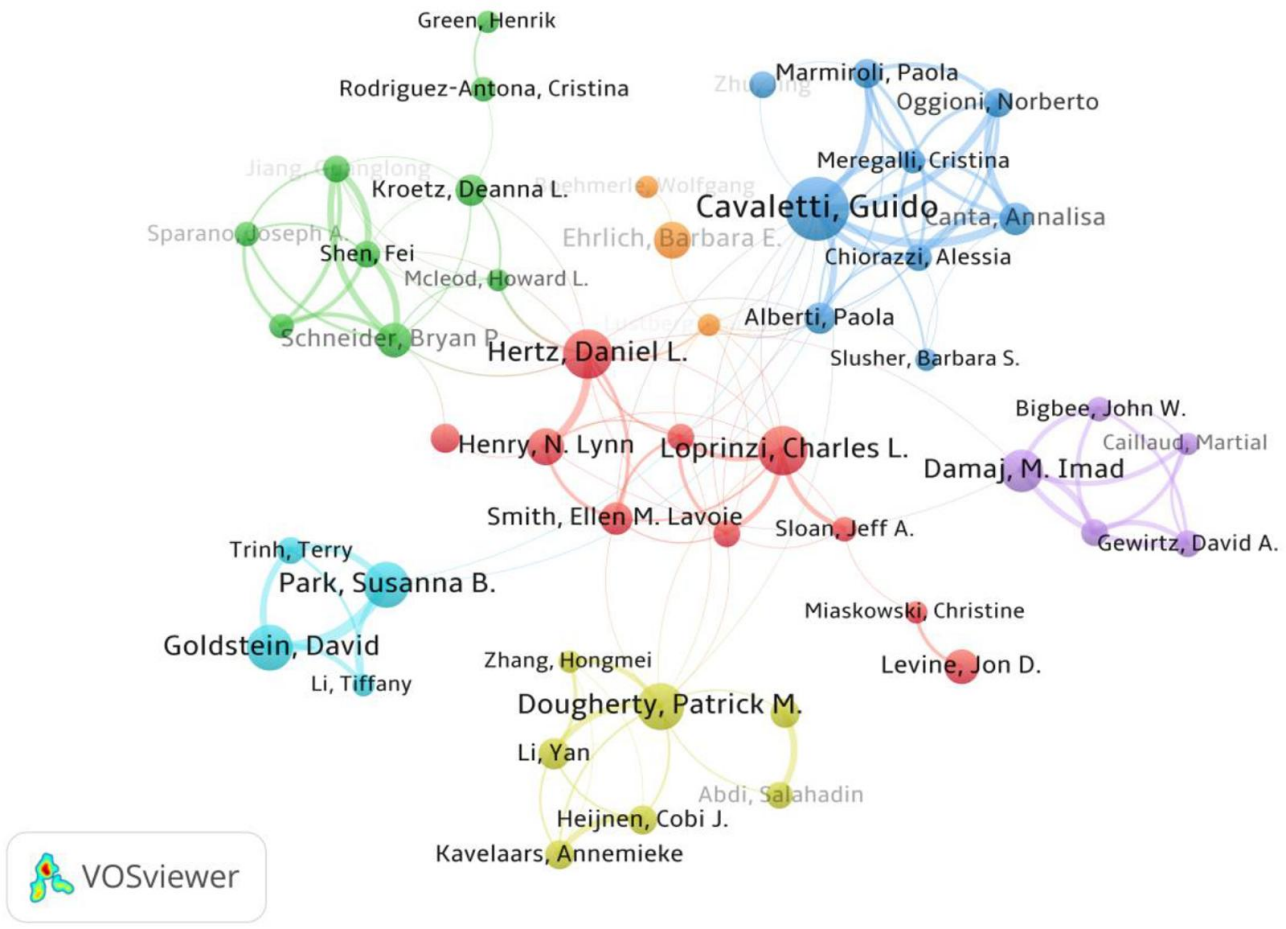
Visualization of research networks of authors.

Identifying prolific authors and their collaborative networks is crucial for understanding the core intellectual structure of this research field. A total of 8,762 authors have contributed to the literature focusing on paclitaxel-induced neuropathic pain. Figure 5 depicts the co-authorship network among core researchers with distinct node colors indicating different collaborative clusters. The visualization reveals several closely connected academic communities. These include a prominent blue cluster centered on Guido Cavaletti which is mainly composed of Italian research teams a red cluster led by Charles L. Loprinzi and Daniel L. Hertz demonstrating close collaboration among U.S. institutions and a cyan cluster represented by David Goldstein and Susanna B. Park which reflects strong academic connections among Australian scholars. Table 4 presents the top 10 authors ranked by publication output. Guido Cavaletti from the University of Milano-Bicocca in Italy stands out as the most productive scholar in this field with 31 publications to his credit. He also boasts the highest Total Link Strength TLS of 69 which indicates his pivotal role as a central hub and bridge in the global collaborative network. Charles L. Loprinzi and Daniel L. Hertz tie for second place each having published 21 articles. In terms of academic impact David Goldstein though ranking fifth in productivity with 19 articles attained the highest Total Citations TC of 5,764. His Average Citations per Publication Avg. citations reached an impressive 303.37 which significantly outperformed those of all other top authors and underscored his profound academic authority and widespread recognition in the field. Patrick M. Dougherty also exhibited exceptional academic impact with an average of 98.75 citations per paper. In contrast certain prolific authors including N. Lynn Henry and Daniel L. Hertz had relatively lower average citation counts of 18.14 and 21.48 respectively. This indicates variations in scholarly attention across different research sub-themes within the field.

**Table 4:** Top 10 authors ranked by number of publications.

Rank	Author	Publications (NP^a^)	Total citations (TC^b^)	TLS^c^	Avg. citations^d^
1	Cavaletti, Guido	31	1,840	69	59.35
2	Loprinzi, Charles L.	21	1,610	29	76.67
3	Hertz, Daniel L.	21	451	35	21.48
4	Dougherty, Patrick M.	20	1,975	31	98.75
5	Goldstein, David	19	5,764	31	303.37
6	Park, Susanna B.	19	646	33	34
7	Damaj, M. Imad	17	552	33	32.47
8	Henry, N. Lynn	14	254	21	18.14
9	Ehrlich, Barbara E.	14	453	2	32.36
10	Levine, Jon D.	13	264	5	20.31

a NP, number of publications.

b TC, total citations.

c TLS, total link strength, a measure of co-authorship network intensity.

d Avg. Citations, average citations per publication.

### 3.6 Co-occurrence of keywords

**Figure 6:**
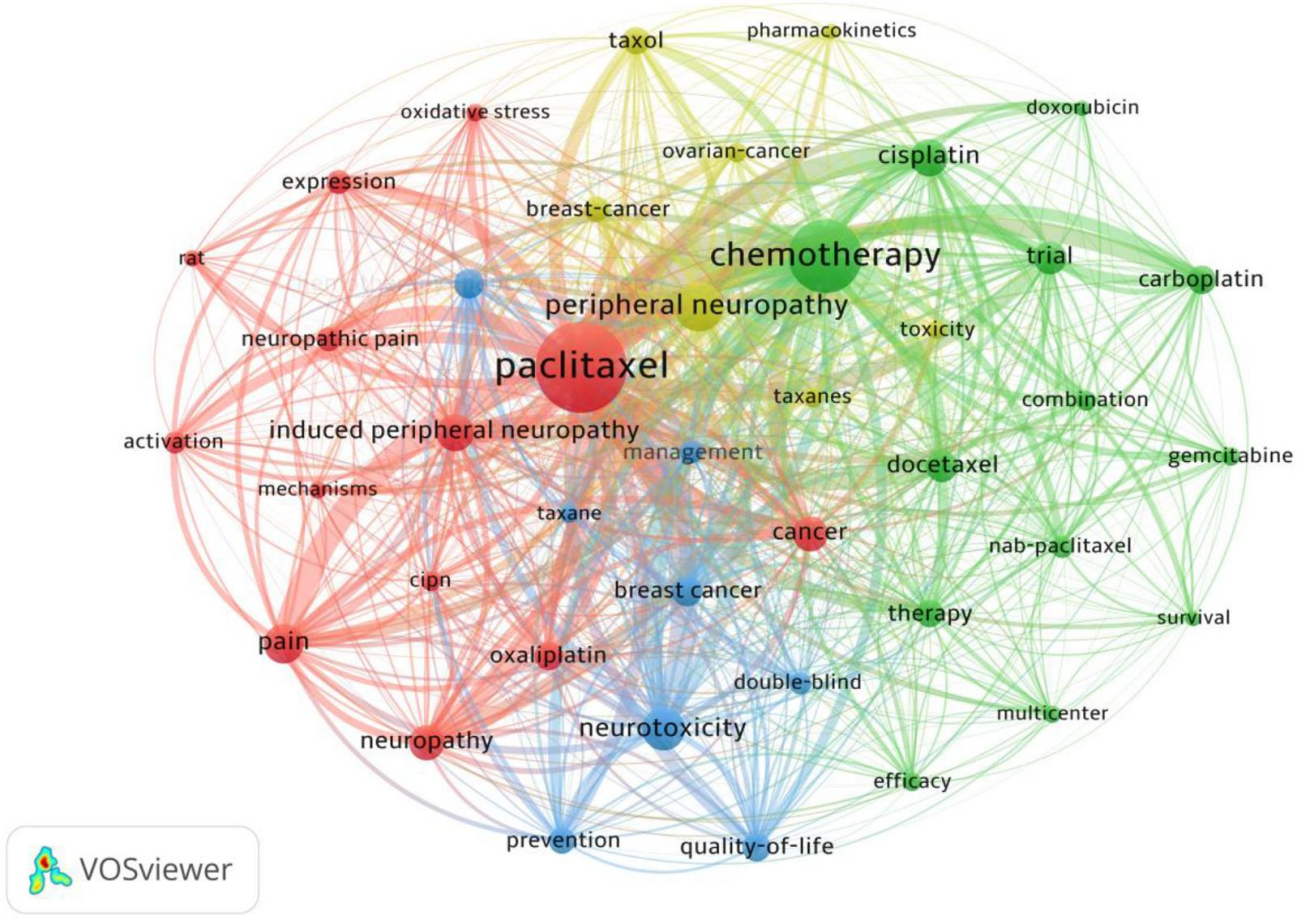
Keyword co-occurrence network.

Identifying prolific authors and their collaborative networks is crucial for understanding the core intellectual structure of this research field. A total of 8,762 authors have contributed to the literature focusing on paclitaxel-induced neuropathic pain. Figure 5 depicts the co-authorship network among core researchers with distinct node colors indicating different collaborative clusters. The visualization reveals several closely connected academic communities. These include a prominent blue cluster centered on Guido Cavaletti which is mainly composed of Italian research teams a red cluster led by Charles L. Loprinzi and Daniel L. Hertz demonstrating close collaboration among U.S. institutions and a cyan cluster represented by David Goldstein and Susanna B. Park which reflects strong academic connections among Australian scholars. Table 4 presents the top 10 authors ranked by publication output. Guido Cavaletti from the University of Milano-Bicocca in Italy stands out as the most productive scholar in this field with 31 publications to his credit. He also boasts the highest Total Link Strength TLS of 69 which indicates his pivotal role as a central hub and bridge in the global collaborative network. Charles L. Loprinzi and Daniel L. Hertz tie for second place each having published 21 articles. In terms of academic impact David Goldstein though ranking fifth in productivity with 19 articles attained the highest Total Citations TC of 5,764. His Average Citations per Publication Avg. citations reached an impressive 303.37 which significantly outperformed those of all other top authors and underscored his profound academic authority and widespread recognition in the field. Patrick M. Dougherty also exhibited exceptional academic impact with an average of 98.75 citations per paper. In contrast certain prolific authors including N. Lynn Henry and Daniel L. Hertz had relatively lower average citation counts of 18.14 and 21.48 respectively. This indicates variations in scholarly attention across different research sub-themes within the field. As shown in Figure 6, the keyword co-occurrence network visually presents high-frequency terms and their internal connections in this field.

### 3.7 Keyword cluster analysis

**Figure 7:**
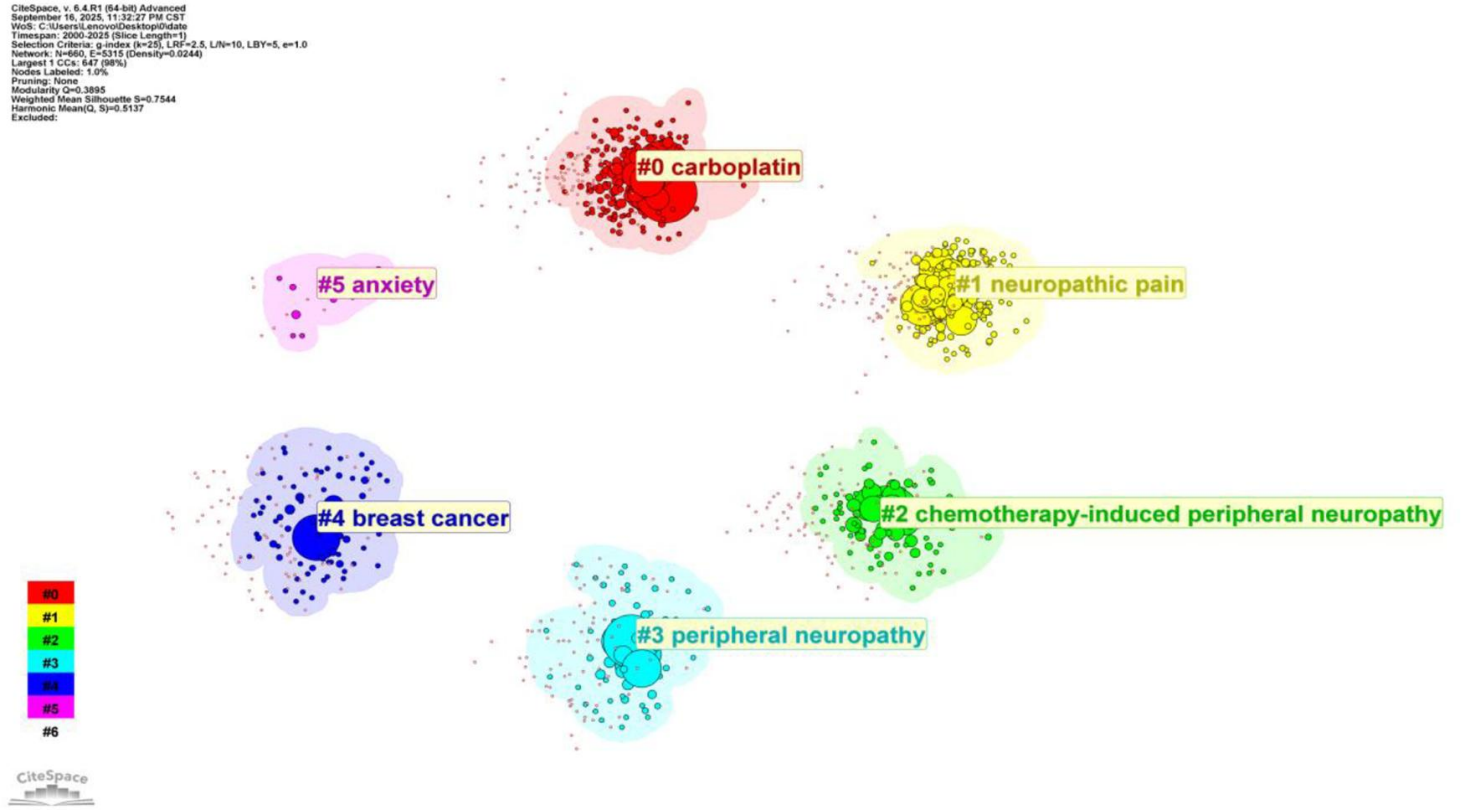
Keyword cluster network.

Keyword cluster analysis functions as a pivotal tool for delineating the distribution patterns and intrinsic associations of research themes within a specific academic domain. In the present study, the Log-Likelihood Ratio (LLR) algorithm integrated in CiteSpace software was employed to generate a keyword clustering knowledge map, which visualized the thematic structure of CIPN research (Figure 7). Network analysis results showed a Modularity Q score of 0.3895 and a Weighted Mean Silhouette S score of 0.7544. Notably, a Q value greater than 0.3 denotes a distinct community structure within the network, whereas an S value exceeding 0.7 indicates high homogeneity and reliability of the clustering results. Consequently, the cluster structure identified in this study exhibits robust credibility (31). Six distinct clusters were visualized through the knowledge map, which concisely encapsulate the current research landscape of the field. Cluster #0, centered on carboplatin, represents the largest cluster. It highlights that the combination of paclitaxel and carboplatin, a standard first-line chemotherapeutic regimen for ovarian and non-small cell lung cancers, is a prominent area of research focus. This cluster encompasses investigations into the synergistic antitumor efficacy of this doublet regimen as well as the cumulative neurotoxic effects associated with their concurrent administration. Clusters #1 (neuropathic pain), #2 (chemotherapy-induced peripheral neuropathy), and #3 (peripheral neuropathy) are closely interconnected and constitute the core thematic focus of the field. These clusters cover an extensive research scope that spans the definition of these neuropathic conditions, clinical symptom evaluation, and the characterization of underlying neuropathological alterations. Cluster #4 focuses on breast cancer, emphasizing that breast cancer patients represent the primary study population in paclitaxel-induced neurotoxicity research. This observation aligns with paclitaxel's established role as a cornerstone agent in adjuvant chemotherapy for breast cancer. Cluster #5, centered on anxiety, represents a relatively distinct and emerging research focus of considerable significance. It reflects a paradigm shift in the field, with research attention expanding from purely physiological mechanisms to a biopsychosocial framework. Specifically, this cluster highlights the increasing scholarly focus on affective comorbidities such as anxiety and depression associated with CIPN, as well as their regulatory effects on chronic pain perception. This trend suggests that a holistic mind-body approach may emerge as a promising direction in future CIPN management strategies.

## 4 Discussion

### 4.1 Principal findings

By robustly integrating data from the Web of Science and Scopus databases, this study presents the first systematic panoramic visualization of the research landscape surrounding chemotherapy-induced neuropathic pain over the past 25 years. First and foremost, the exponential growth in research output provides compelling evidence for the growing clinical relevance of this field. With advances in early cancer diagnosis and the widespread adoption of potent chemotherapeutic agents such as paclitaxel have significantly prolonged patient survival, long-term sequelae, particularly neuropathic pain, have become major bottlenecks that limit the quality of life of cancer survivors (32). This trend indicates a profound paradigm shift in oncology practice, which has moved from an exclusive focus on tumor eradication to a comprehensive emphasis on quality of survivorship. Second, the global research landscape exhibits a pronounced hierarchical structure with the United States at the forefront. Building on its extensive foundational research in basic medicine and the abundant resources of top-tier clinical centers including MD Anderson Cancer Center and the Mayo Clinic, the United States maintains an absolute leading position in terms of publication volume, total citations and network centrality. In contrast, although China ranks third in research productivity with a robust upward trend in output, its average citation count (21.25) remains substantially lower than that of Western countries and Australia (152.98). This discrepancy suggests that research in developing nations may tend to prioritize confirmatory clinical observations or single-center research, which may limit their international academic influence. Consequently, there is an urgent need to enhance academic impact through strengthening international multi-center collaborative initiatives and advancing high-quality translational research projects. Third, the evolution of research hotspots reflects a clear paradigm shift from phenomenological description to mechanistic elucidation and, ultimately, to comprehensive management strategies. Early research primarily centered on documenting the incidence and clinical manifestations of paclitaxel-induced neurotoxicity. Subsequently, the high frequency of keywords such as oxidative stress and mitochondrial dysfunction indicated a growing emphasis on unraveling the underlying molecular mechanisms (33). Most importantly, the emergence of Cluster #5 (anxiety) marks a pivotal cognitive advancement in the field. It represents a move away from a purely biomedical model centered exclusively on somatic pain toward a biopsychosocial framework that encompasses the interactions between pain and emotional states (34). This transition implies that future clinical management strategies must integrate psychological intervention approaches for comorbid emotional disorders alongside targeted nerve repair therapies. Finally, despite the accumulating body of research findings, prevention remains a high-frequency keyword that reflects an unresolved clinical dilemma. The current absence of FDA-approved prophylactic agents for chemotherapy-induced neuropathic pain (35) highlights a persistent translational gap between basic mechanistic research and clinical application. This gap underscores the urgent need for breakthroughs through precision medicine strategies including pharmacogenomic screening to identify susceptibility phenotypes, which could enable personalized prevention and intervention approaches for high-risk patients.

### 4.2 Limitations

Several limitations must be acknowledged when interpreting the findings of this study. First, inherent language bias is inevitable, given that restricting the analysis to English-language publications may have inadvertently excluded high-quality research published in native-language journals from non-English-speaking countries. This oversight could introduce gaps in the overall research landscape we evaluated, as valuable insights from non-English literature might be overlooked. Second, while Web of Science and Scopus are recognized as the most comprehensive academic databases currently available, incomplete data coverage cannot be fully ruled out. Specifically, some emerging or highly specialized niche journals may not be indexed in these two platforms, which could result in the omission of relevant studies that might otherwise provide meaningful contributions to the field. Finally, bibliometric indicators are inherently subject to a time-lag effect. As citation accumulation is a time-dependent process, recent high-quality studies published between 2024 and 2025 may be underestimated in the current citation analysis; this could potentially obscure their true academic impact in the long term, as their citation counts have not yet had sufficient time to accumulate to a stable and representative level.

## 5 Conclusions

In conclusion, research on paclitaxel-induced neuropathic pain is currently in a “golden age” of rapid development and expansion. The United States and its leading cancer research institutions have established global dominance in this field, whereas emerging powers represented by China are advancing rapidly. The research focus has progressively deepened from the initial description of toxicological phenomena to the elucidation of key molecular mechanisms, including oxidative stress and neuroinflammation. It is now further expanding toward the development of comprehensive management models that incorporate the management of psychological comorbidities. Looking ahead, the key breakthrough in this field will lie in bridging the gap between basic mechanistic research and clinical translation. Future research efforts should prioritize three key directions: first, Translational Medicine, which aims to translate identified molecular targets into effective neuroprotective strategies and agents; second, Precision Medicine, leveraging genomic technologies to identify high-risk populations and guide personalized dosing regimens; third, Holistic Intervention, focusing on the development of multimodal management protocols that integrate pharmacotherapeutic approaches with psychological support interventions. This study provides a comprehensive knowledge mapping and navigation framework for clinicians and researchers. It is anticipated to facilitate the optimization of research resource allocation and accelerate progress toward overcoming this persistent clinical challenge.

## Data Availability

The original contributions presented in the study are included in the article/Supplementary Material, further inquiries can be directed to the corresponding author.
